# High expression of oncogene cadherin-6 correlates with tumor progression and a poor prognosis in gastric cancer

**DOI:** 10.1186/s12935-021-02071-y

**Published:** 2021-09-16

**Authors:** Zongxian Zhao, Shuliang Li, Shilong Li, Jun Wang, Hai Lin, Weihua Fu

**Affiliations:** 1 Tianjin Medical University General Hospital, No. 154 Anshan Road, Heping District, Tianjin, China; 2 Department of Gastrointestinal Surgery, The Second People’s Hospital of Liaocheng, Liaocheng, Shangdong China

**Keywords:** Cadherin-6, Gastric cancer, Tumor progression, Prognosis, Oncogene

## Abstract

**Background:**

Gastric cancer (GC) is one of the most common and fatal cancers worldwide. Effective biomarkers to aid the early diagnosis of GC, as well as predict the course of disease, are urgently needed. Hence, we explored the role and function of cadherin-6 (CDH6) in the diagnosis and prognosis of gastric cancer.

**Methods:**

The expression levels of CDH6 in cancerous and normal gastric tissue were analyzed using multiple public databases. Gene set enrichment analysis (GSEA) was performed using The Cancer Genome Atlas (TCGA) dataset. The diagnostic efficiency of CDH6 expression in GC patients was determined through receiver operating characteristic (ROC) curve analysis. The associations between clinical variables and CDH6 expression were evaluated statistically, and the prognostic factors for overall survival were analyzed by univariate and multivariate Cox regression. 44 GC tissue samples, 20 donor-matched adjacent normal tissue samples, and associated detailed clinical information, were collected from the Tianjin Medical University General Hospital. CDH6 expression levels were determined for further validation.

**Results:**

CDH6 was upregulated in GC samples compared to normal gastric tissue. Furthermore, GSEA identified the tricarboxylic acid (TCA) cycle, extracellular matrix (ECM) receptor interaction, glyoxylate and dicarboxylate metabolism, oxidative phosphorylation, and the pentose phosphate pathway as differentially enriched in GC tissue samples. According to the area under the ROC curve (AUC) values (AUC = 0.829 in the TCGA and 0.966 in the GSE54129 dataset), CDH6 expression was associated with high diagnostic efficacy. Patients with high CDH6 levels in their GC tissues had a higher T number (according to the TNM classification) and a worse prognosis than those with low CDH6 expression. Univariate and multivariate Cox regression analysis showed that CDH6 was an independent risk factor for overall survival (univariate: HR = 1.305, P = 0.002, multivariate: HR = 1.481, P < 0.001).

**Conclusion:**

CDH6 was upregulated in GC, and high CDH6 expression was indicative of a higher T number and a worse prognosis. Therefore, CDH6 represents a potentially independent molecular biomarker for the diagnostic and prognostic prediction of GC.

**Supplementary Information:**

The online version contains supplementary material available at 10.1186/s12935-021-02071-y.

## Background

Gastric cancer (GC) is one of the most common and lethal cancers worldwide [1]. It is the third most-common type of cancer and the second common cause of cancer-related deaths in China [2]. Advances in surgical techniques, traditional radiotherapy, chemotherapy, and the implementation of neoadjuvant therapy, have collectively improved the treatment of early GC [3]. However, due to the nonspecific symptoms of GC, most patients with the disease are diagnosed only when the tumor has reached an advanced stage, at which point the disease has likely metastasized and is inoperable, leading to a poor prognosis and low 5-year overall survival rates [4, 5]. Therefore, in order to increase the long-term survival of patients, further research is urgently needed to identify highly sensitive specific biomarkers for the early and accurate diagnosis of GC.

Cadherins (CDHs) are a multigene family of proteins that mediate homophilic calcium-dependent cell adhesion. CDHs play critical roles in morphogenesis by mediating specific intercellular adhesion and organization of the cytoskeleton [6]. In addition, CDHs can also serve as sensors of the surrounding microenvironment and as signaling centers for cellular pathways [7]. Recently, several studies have found that CDHs can participate in the promotion of tumorigenesis, tumor growth, and malignant progression, and can be exploited for the diagnosis and survival prediction or cancer patients, and even as therapeutic targets [8–10]. For example, the transcriptional silencing or mutation of E-cadherin is correlated with familial diffuse GC, which may serve as a biomarker for early cancer diagnosis [11]. It has also been shown that CDH2 could act as a potential prognostic and predictive biomarker for the grading and treatment of gliomas [12].

Cadherin-6 (CDH6) is a class II CDH, mainly involved in the morphogenesis of the central nervous system and kidney [13, 14]. CDH6 contains five extracellular domains and a large cytoplasmic domain, which it uses for interacting with catenin molecules. CDH6 also contains RGD motifs and the His-Ala-Val (HAV) motif for the stabilization and clustering of adjacent monomers at the five extracellular domains, which sets it apart from other cadherin family members such as CDH1, CDH2, or CDH3 [15]. It is unknown whether its special structures could play a specific role in regulating biological function in occurrence and development of tumors. Previous studies have reported that CDH6 could be abnormally upregulated and promote epithelial mesenchymal transition (EMT) and cancer metastasis by attenuating autophagy in the context of papillary thyroid carcinomas [16, 17]. In addition, increased CDH6 expression has been reported in several malignancies (including nasopharyngeal, ovarian, oral squamous cell, and renal cancers) and is associated with lymph node metastases and a poor prognosis [18–20]. Sotomayor et al*.* proposed that *CDH6* may act as a lineage gene, with its expression being maintained in some tumors [21]. Furthermore, it was reported that CDH6 could cause tumor cells to lose cellular polarity, further highlighting the potential for CDH6 as a target for antibody–drug conjugate (ADC) therapy development [22]. Encouragingly, it was reported that HKT288, a CDH6-targeting ADC, could cause tumor regression in ovarian and renal cancer [23]. However, the clinical significance, as well as the diagnostic and prognostic value of CDH6 in GC remain unclear. Further investigations are required to understand whether CDH6 could be used as a novel biomarker for the diagnosis, prognosis prediction, and treatment of GC. In this report, we provide a comprehensive and systematic analysis of CDH6 expression in GC tissues as compared to normal gastric tissues. To further study the function of CDH6, we used Gene Set Enrichment Analysis (GSEA) to evaluate the biological pathways involved in GC pathogenesis. Survival analyses (Cox regression analyses) were also performed to assess the prognostic value of CDH6 expression alongside other clinicopathological features.

## Methods

### Data collection

The gene expression profiles and associated clinicopathological data belonging to patients with gastric adenocarcinoma were downloaded from TCGA Genomic Data Commons Data Portal (https://portal.gdc.cancer.gov/repository) on the 25th May 2020. RNA-Seq gene expression HTSeq-FPKM data for 343 cancer tissue samples and 30 normal, adjacent tissue samples were collected for further analysis. To ensure the accuracy of TCGA results, we systematically retrieved the GEO (Gene Expression Omnibus) microarray, and five datasets (GSE50710, GSE70880, GES109476, GSE118916, GSE54129) were obtained. Oncomine (http://www.oncomine.org), a web-based microarray tool, was used to analyze the expression level of CDH6 in gastric cancer tissues and normal control samples.

### Gene set enrichment analysis

Gene set enrichment analysis (GSEA) is a computational method used to detect whether a priori defined gene sets have statistically significant and consistent differences between two biological states [24]. Datasets and phenotype label files from TCGA were generated and uploaded onto the GSEA software. The phenotype labels were CDH6-high expression and CDH6-low expression. Gene set permutations were conducted 1000 times for each analysis. Gene sets with ES > 0.6, FWER P values < 0.05 were considered as enriched.

### Cell culture and clinical samples

The GC cell line NCI-N87 was purchased from the National Experimental Cell Resource Sharing Platform (Beijing, China). The GC cell lines HGC-27 and MGC-803, and one normal gastric epithelial cell line (GES-1), were collected form the Laboratory of General surgery, Tianjin Medical University General Hospital (Tianjin, China). Cells were cultured in 1640 medium (Thermo Fisher Scientific, Waltham, MA, USA) containing 10% FBS (Thermo Fisher Scientific), 80 U mL^−1^ penicillin and 0.08 mg mL^−1^ streptomycin under a humidified atmosphere with 5% CO_2_ at 37 °C. The culture medium was replaced every 48 h. The cells were screened periodically for mycoplasma contamination using the One-step Quickcolor Mycoplasma Detection Kit (Shanghai, China). 44 GC and 20 normal adjacent tissue samples were collected from GC patients at the Tianjin Medical University General Hospital. Corresponding clinical characteristics (patient age, cancer stage and grade, distant metastasis status, lymph node status, survival time, and survival status) were also collected and analyzed. The patients were classified into two groups: individuals under 65 years of age (younger group); or those aged 65 or older (older group). Written informed consent was obtained from all patients; the hospital ethics review committees approved this study.

### Quantitative real-time polymerase chain reaction

Total RNA was extracted from cells using the RNAprep Pure Tissue kit (Tiangen, Beijing, China). Next, complementary DNA (cDNA) was reverse synthesized using the FastKing gDNA Dispelling RT SuperMix for qPCR (Tiangen, Beijing, China). Quantitative real-time polymerase chain reaction (qRT-PCR) was performed using the 2 × SYBR Green qPCR Master Mix (Tiangen, Beijing, China). The relative mRNA expression levels were calculated using the 2^−ΔΔCt^ method. We used the average expression of CDH6 in 20 normal gastric tissues as a reference for normalization and comparison to the 20 gastric tumor tissues. The primer sequences were as follows: CHD6, forward (5′-TAT CAG ACC CCG ACC ATA TT-3′) and reverse (5′-GAC CAT AAA CTT CCG GCT T-3′); β-actin, forward (5′-CTC CTC CAC CTT TGA CGC TG-3′) and reverse (5′-TCC TCT TGT GCT CTT GCT GG-3′). All gene primers were obtained from Aoke Dingsheng Biotechnology (Beijing, China). The thermocycling conditions comprised an initial denaturation at 95 °C for 15 s, followed by 40 cycles of 53 °C for 30 s and 72 °C for 30 s.

### Survival and Cox regression analyses

We divided TCGA samples into two groups by the median value of *CDH6* gene expression to construct the survival curve. Univariate and multivariate Cox analyses were used to investigate the role of CDH6 expression and other clinical characteristics (age, cancer stage and grade, distant metastasis status, and lymph node status) in overall survival. In addition, survival analysis was directly verified using the Kaplan–Meier plotter (http://kmplot.com/).

### Statistical analysis

R3.5.2, Bioconductor (https://www.bioconductor.org/), and GraphPad Prism 8 were used for statistical analysis. Survival curves were plotted using the Kaplan–Meier method and were compared using the log-rank test. Cox regression analyses were completed using the R ‘survival’ package. The relationship between clinical pathologic features and CDH6 expression were completed using the Wilcoxon signed rank test and the Kruskal–Wallis test. P < 0.05 was considered as an indicator of statistical significance.

## Results

### CDH6 is highly expressed in GC

**Fig. 1:**
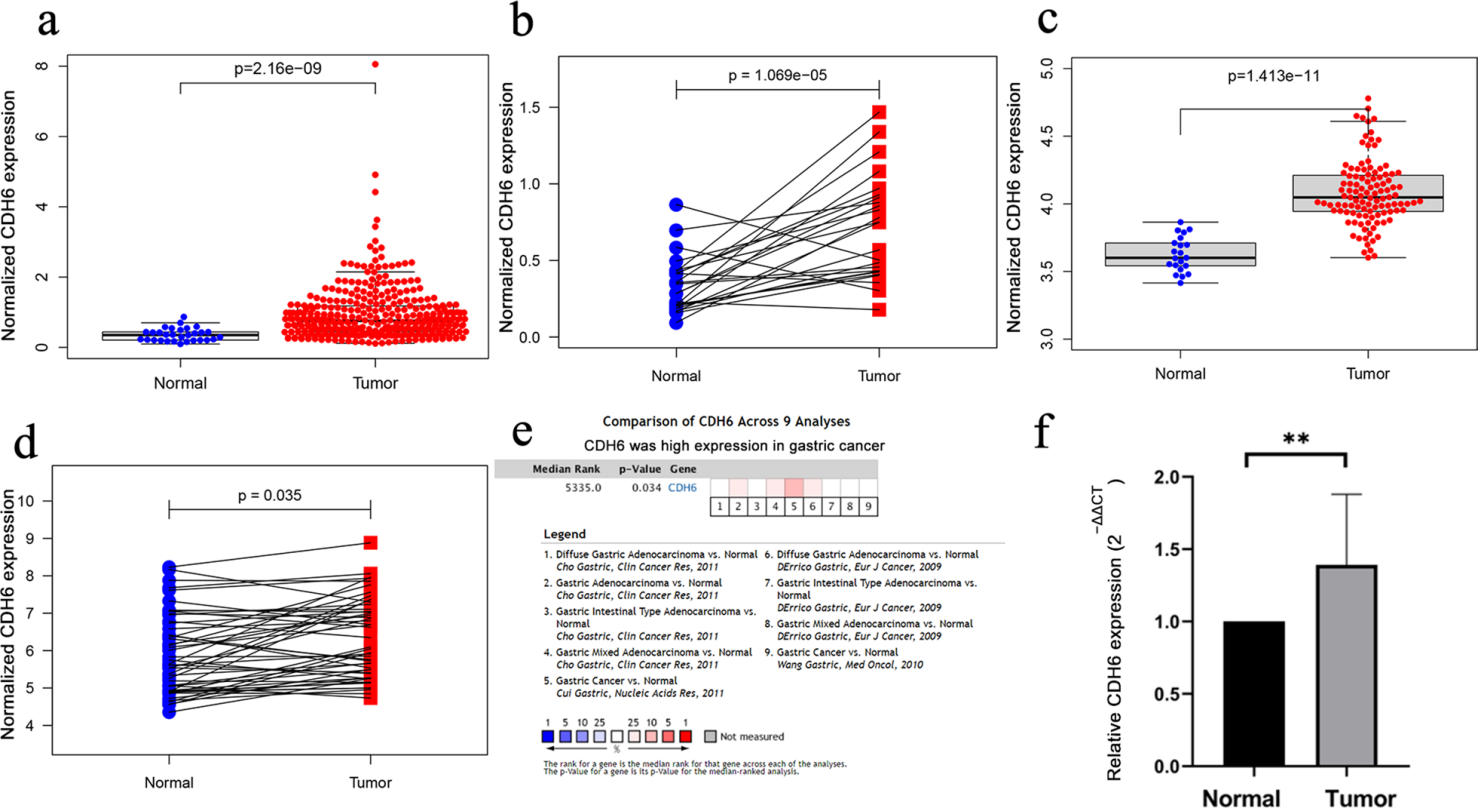
CDH6 expression levels in cancerous and normal gastric tissues. **a** CDH6 expression in 343 GC tissues and 30 normal tissues, from the TCGA database. Wilcoxon signed rank test P = 2.16e−09. **b** CDH6 expression in 25 GC tissues and donor-matched normal tissues from TCGA. Wilcoxon matched pairs test P = 1.069e−05. **c** CDH6 expression in 111 GC tissues and 21 healthy volunteer-derived tissues from GEO (GSE54129). Wilcoxon signed rank test P = 1.413e−11. **d** CDH6 expression in 50 GC tissues and donor-matched normal tissues from GEO (GSE50710, GSE70880, GES109476, and GSE118916). Wilcoxon matched pair test P = 0.035. **e** Meta-analysis of CDH6 expression using the Oncomine analysis tool P = 0.034. **f** CDH6 expression in cancerous tissues from 20 GC patients and donor-matched normal controls (from the Tianjin Medical University General Hospital cohort). The average expression of CDH6 in the 20 donor-matched normal gastric tissue samples was regarded as a reference. Paired Student’s *t*-test P = 0.0019

**Fig. 2:**
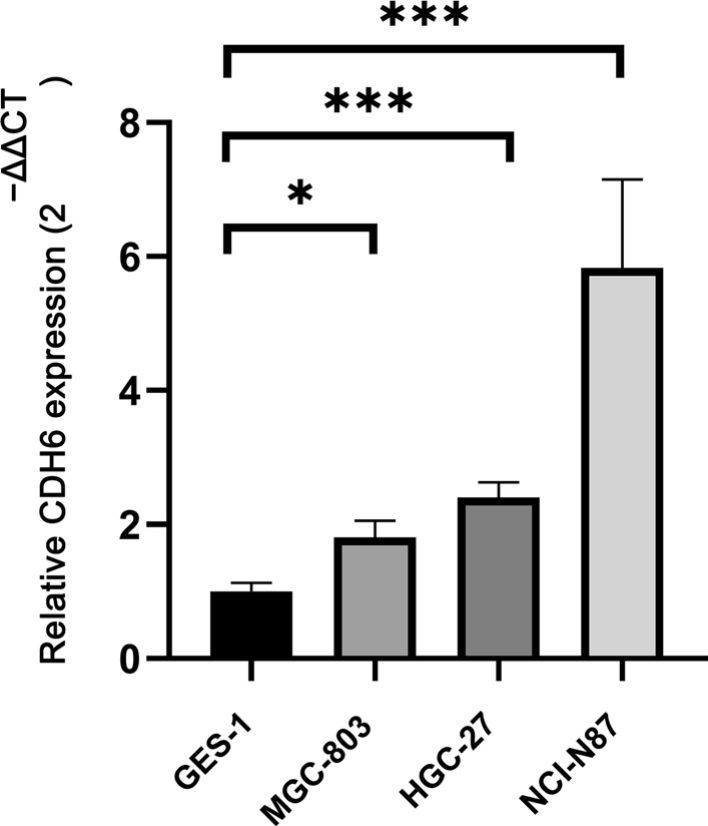
CDH6 expression in normal gastric epithelial cell lines (GES-1) and three GC cell lines (HGC-27, MGC-803, and NCI-N87) by qRT-PCR. One-way ANOVA test *P < 0.05, **P < 0.01, ***P < 0.001

According to TCGA, CDH6 was highly expressed in 343 GC tissues as compared to the 30 normal tissues (P = 2.16e−09, Fig. 1a) and in 25 GC tissues compared to the donor-matched normal tissues (P = 1.069e−05, Fig. 1b). Meanwhile, we analyzed the differential expression of CDH6 in 111 GC tissues compared with 21 normal gastric tissues obtained from healthy volunteers, using the GSE54129 dataset (P = 1.413e−11, Fig. 1c), and in 50 GC tissues compared with donor-matched normal gastric tissues from four other datasets (GSE50710, GSE70880, GES109476, and GSE118916) (P = 0.035, Fig. 1d). According to the Oncomine, CDH6 was upregulated in GC tissues (P = 0.034, Fig. 1e). To further verify CDH6 expression, we measured the expression level of CDH6 in 20 donor-matched GC tissues by qRT-PCR (P < 0.05, Fig. 1f) and in the normal gastric epithelial cell line GES-1, and three GC cell lines (HGC-27, MGC-803, and NCI-N87) (P < 0.05, Fig. 2).

### GSEA identifies functions and signaling pathways

**Fig. 3:**
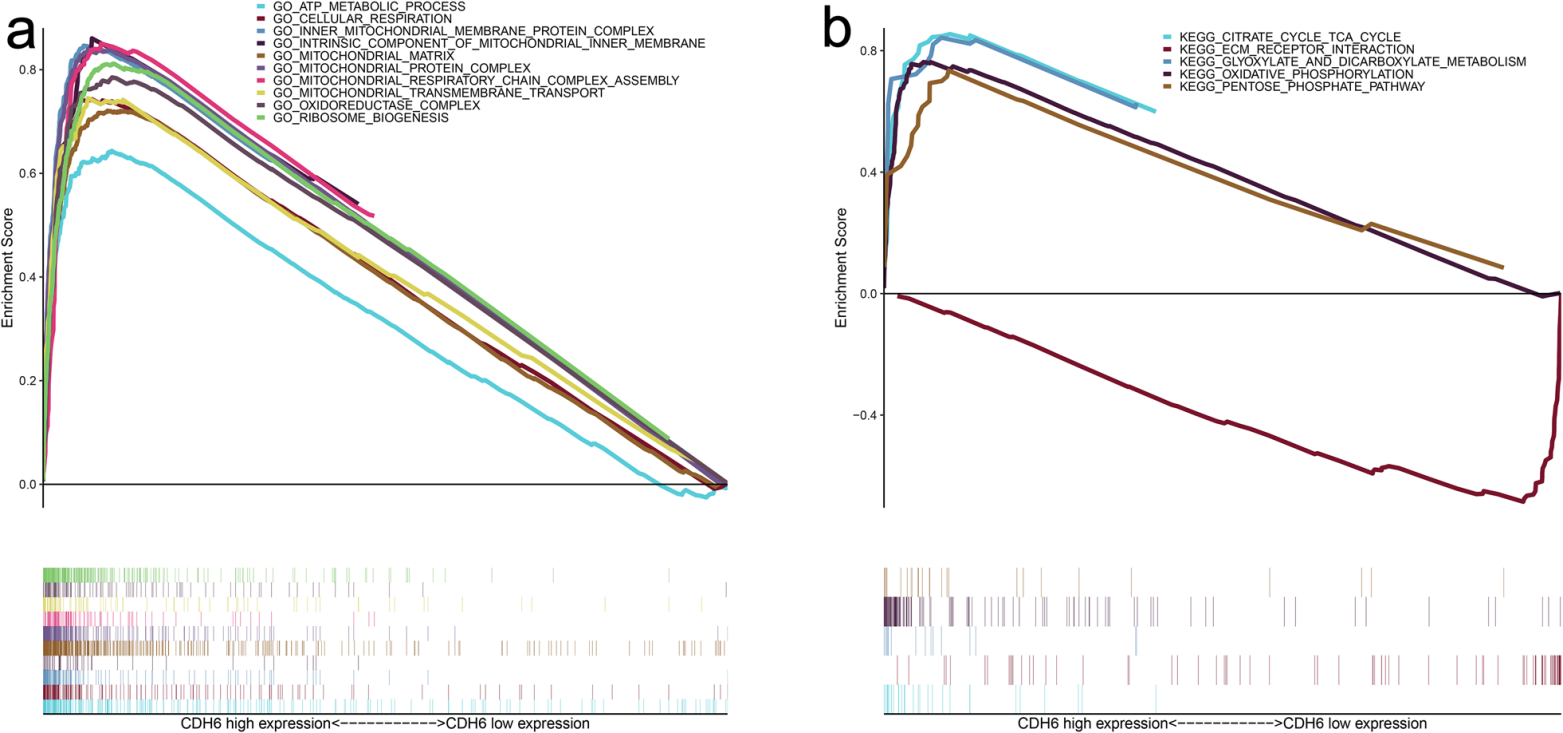
Enrichment plots from the gene set enrichment analysis (GSEA). **a** GSEA results showing differential enrichment of genes in GO with high CDH6 expression. **b** GSEA results showing differential enrichment of genes in KEGG with high CDH6 expression

To analyze the biological characteristics shared by tissue samples displaying different CDH6 expression levels and predict the functions and pathways in which CDH6 may be involved, we performed the GSEA assay. Gene Ontology (GO) enrichment analysis indicated that the most enriched genes were associated with the following processes: ATP metabolic process, cellular respiration, inner mitochondrial membrane protein complex, intrinsic component of the mitochondrial inner membrane, mitochondrial matrix, mitochondrial protein complex, mitochondrial respiratory chain complex assembly, mitochondrial transmembrane transport, oxidoreductase complex, and ribosome biogenesis (Fig. 3a). In addition, Kyoto Encyclopedia of Genes and Genomes (KEGG) analysis found that genes belonging to the following processes: TCA cycle, glyoxylate and dicarboxylate metabolism, oxidative phosphorylation, and pentose phosphate pathway, were significantly enriched for in the CDH6 high-expressing GC samples. On the other hand, ECM receptor interaction correlative genes were significantly in the CDH6 low-expressing GC samples (Fig. 3b).

### CDH6 is of high diagnostic value in GC

**Fig. 4:**
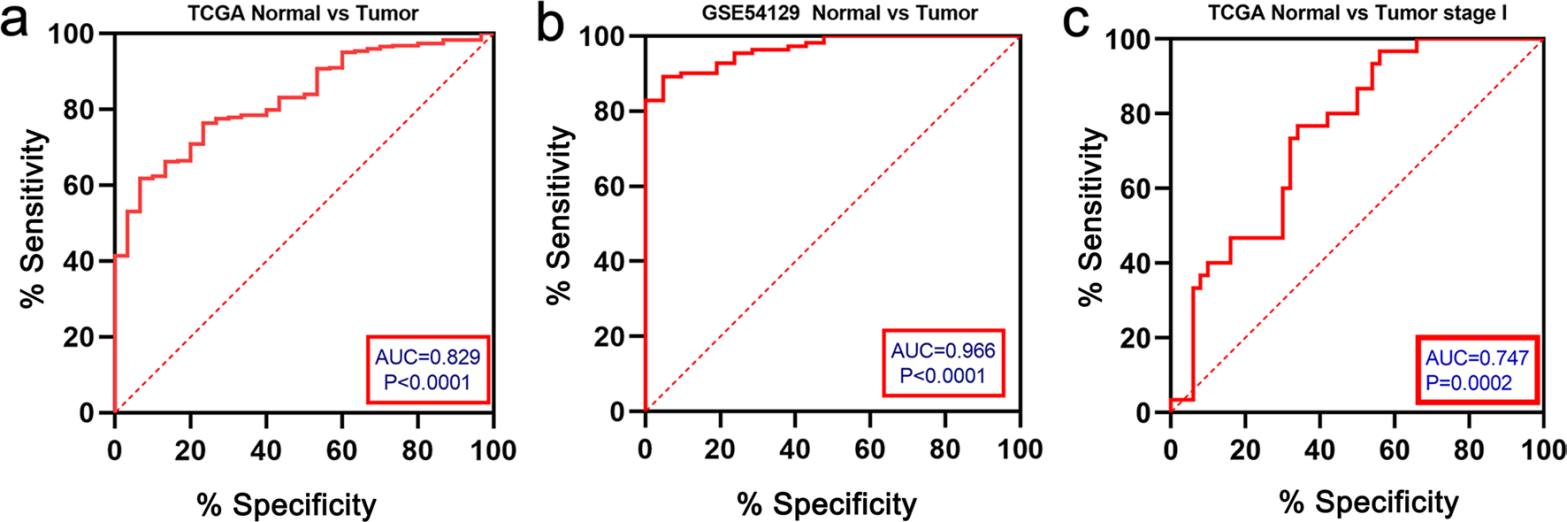
ROC curve for CDH6 expression in normal and cancerous gastric tissue samples. **a** 343 GC tissues vs. 30 normal tissues from TCGA. **b** 111 GC patients versus 21 healthy individuals from the GSE54129 dataset. **c** 50 stage I gastric cancer tissues versus 30 normal tissues from TCGA

To evaluate the diagnostic value of CDH6, the receiver operating characteristic (ROC) curve was constructed using gene expression data from 343 GC and 30 normal tissues, derived from TCGA. The area under the ROC curve (AUC) was 0.829 [95% confidence interval (CI): 76.9–89.0%], the sensitivity was 61.8% (95% CI: 56.6–66.8%), and the specificity was 93.3% (95% CI: 78.7%–98.8%; Fig. 4a). For further verification, we generated another ROC curve using expression data from 111 GC patients and 21 healthy individuals from the GSE54129 dataset. The AUC was 0.966 (95% CI: 0.938–0.994), the sensitivity was 89.2% (95% CI: 0.820–0.937), and the specificity was 95.2% (95% CI: 77.3%–99.8%; Fig. 4b). Collectively, both ROCs indicated the potential diagnostic value of CDH6 in GC. To evaluate the diagnostic value of CDH6 in the early detection of GC, the ROC curve was constructed using gene expression data from 50 stage I and 30 normal tissue samples, derived from TCGA [Fig. 4c (AUC = 0.747, 95% CI: 0.641–0.853, P = 0.0002)].

### High CDH6 expression is associated with tumor progression

**Fig. 5:**
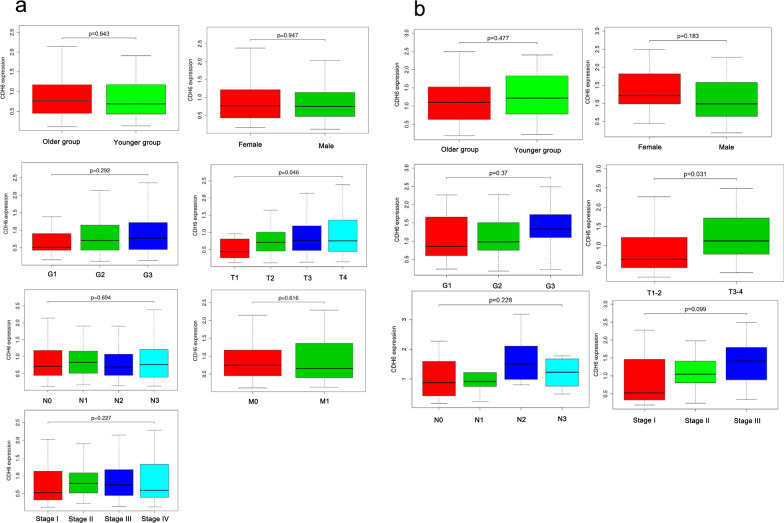
The correlation of the level of CDH6 expression with clinical GC variables from the TCGA database (**a**) or the Tianjin Medical University General Hospital (**b**)

We analyzed the clinical pathologic data relating to 343 patients with GC derived from TGCA, including the patients’ age, sex, clinical stage, histological grade, and tumor-lymph node-metastasis (TNM) classification. As shown in Fig. 5a, the expression of CDH6 was only significantly associated with the T stage TNM classification (P = 0.046). High levels of CDH6 were unrelated to age, sex, clinical stage, histological grade, lymph node metastasis, and distant metastasis. The same analysis outcomes were observed in the 44 GC patients recruited at the Tianjin Medical University General Hospital (T1-2 VS T3-4, P = 0.031; Fig. 5b).

### High CDH6 expression is associated with worse prognosis

**Fig. 6:**
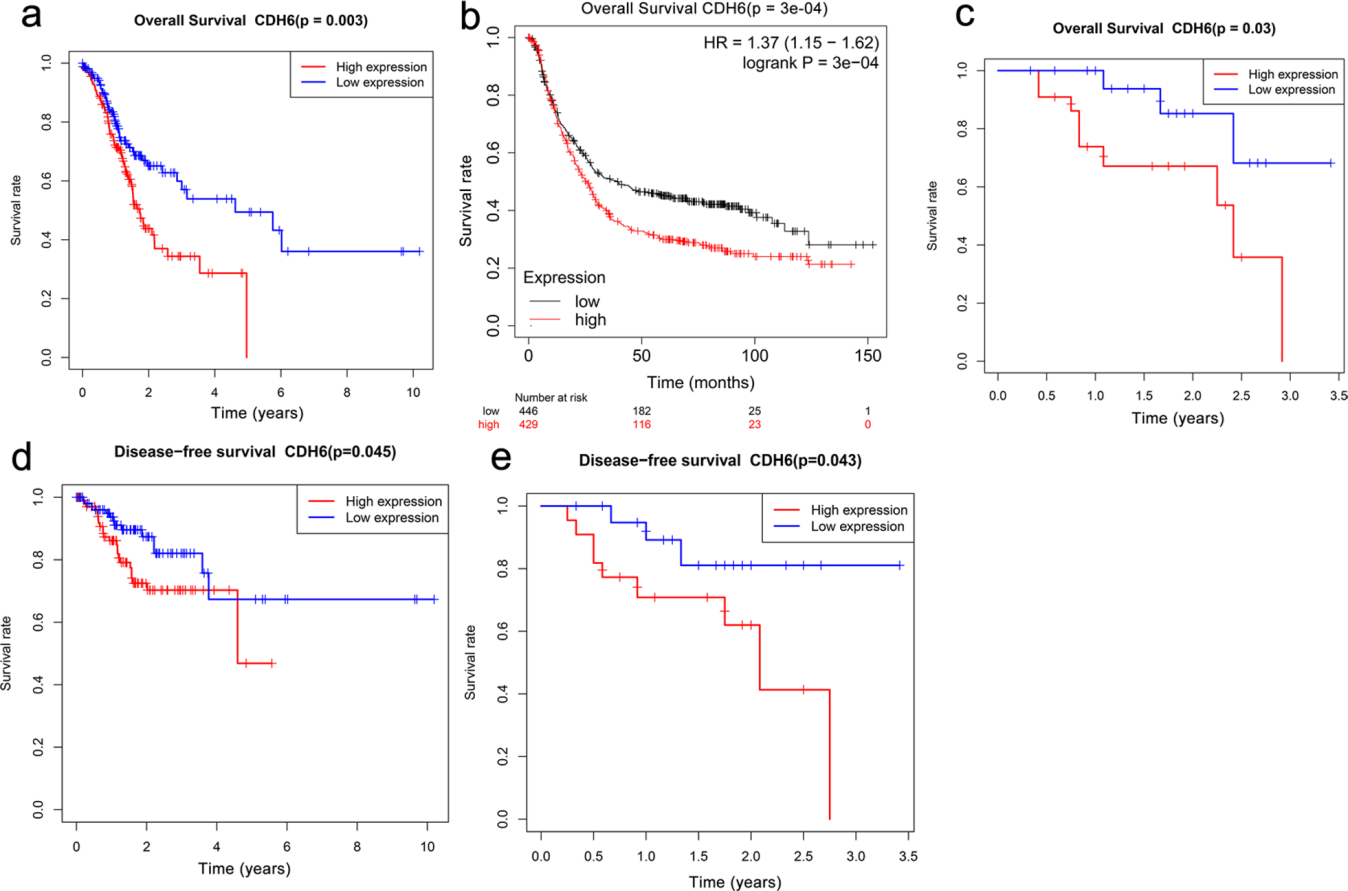
High expression level of CDH6 lead to a poor prognosis in GC. **a** CDH6 expression in and overall survival of GC patients from the TCGA cohort. **b **CDH6 expression and overall survival determined using the Kaplan–Meier plotter. **c** CDH6 expression in and overall survival of the 44 GC patients recruited from the Tianjin Medical University General Hospital. **d** CDH6 expression in and disease-free survival of the TCGA cohort. **e** CDH6 expression in and disease-free survival of the 44 CG patients from the Tianjin Medical University General Hospital cohort

**Fig. 7:**
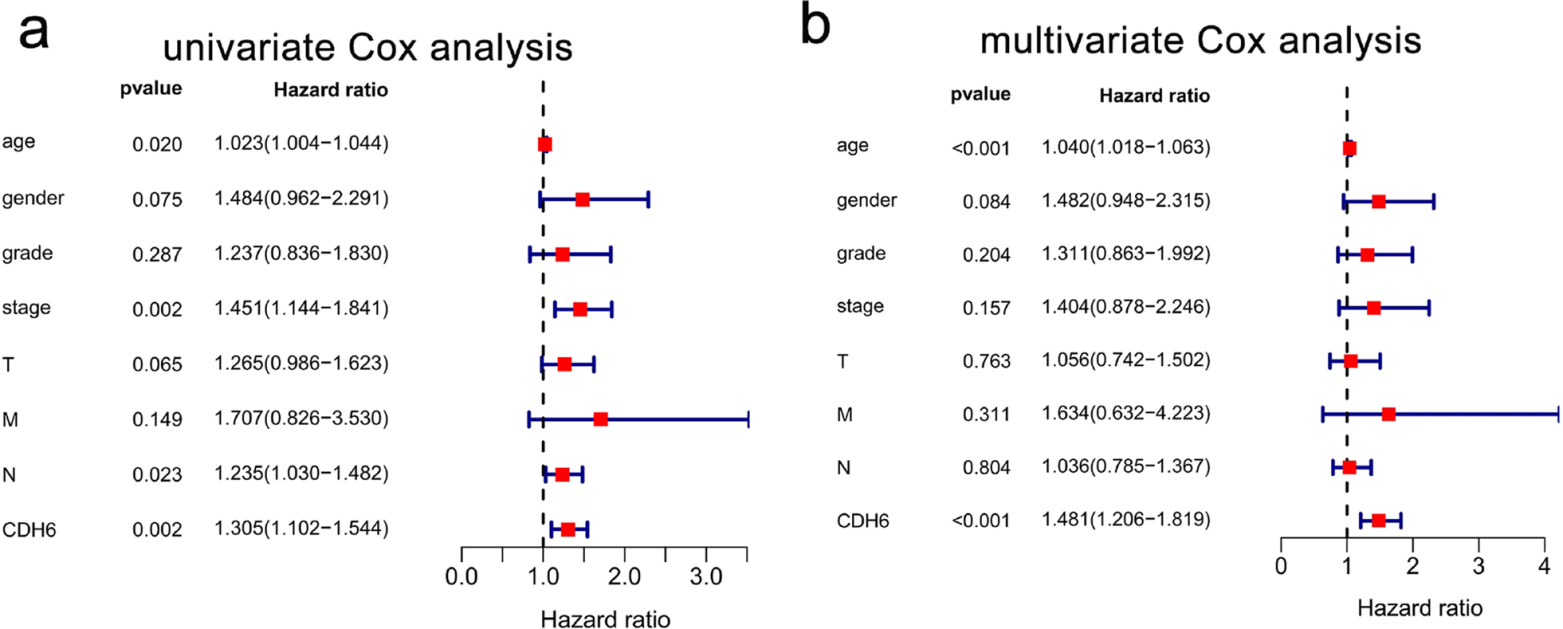
Cox regression analysis of CDH6 expression and clinical pathological characteristics. **a** The univariate Cox analysis. **b** The multivariate Cox analysis

As shown in Fig. 6a, high expression of CDH6 was closely associated with poor overall survival (P < 0.01). This relationship was further validated by the online Kaplan–Meier plotter (http://kmplot.com/; Fig. 6b, P < 0.01). The above results were also confirmed in our cohort of 44 patients with GC (Fig. 6c, P < 0.05). In addition, high expression of CDH6 was also associated with poor disease-free survival according to TCGA data (Fig. 6d, P < 0.05). And the disease-free survival analysis of our 44 patients with GC also confirmed the result (Fig. 6e, P < 0.05). The univariate Cox analysis revealed that high CDH6 expression was significantly associated with poor overall survival [hazard ratio (HR): 1.305, 95% CI: 1.102–1.544, P = 0.002]; as well as age (HR: 1.023, 95% CI: 1.004–1.044, P = 0.020); stage (HR = 1.451, 95% CI: 1.144–1.841, P = 0.002); and N stage (HR = 1.305, 95% CI: 1.102–1.544, P = 0.002) among GC patients (Fig. 7a). Moreover, multivariate Cox analysis indicated that high CDH6 expression remained an independent risk factor for overall survival with an HR of 1.481 (95% CI: 1.206–1.819, P < 0.001), as well as age (HR = 1.040, 95% CI: 1.018–1.063, P < 0.001) among GC patients (Fig. 7b).

## Discussion

The primary function of the CDH family of proteins is in cell–cell and cell–matrix adhesion, which define cellular interactions with the surrounding microenvironment [7]. In cancer, any disfunction in cell–cell and cell–matrix adhesion are related to tumor progression, lymph node infiltration, and distant metastasis [25]. It has been previously shown that tumor growth, malignant progression, and distant metastasis were associated with cellular adhesion molecules such as CDHs, integrins, and immunoglobulins [26, 27]. Several recent studies involving CDHs have indicated that these proteins not only have structural functions but can also regulate complex biological signals and participate in the promotion of tumorigenesis, tumor growth, and malignant progression. For example, the *CDH1* gene is associated with familial diffuse GC and the process of EMT [11, 28]. Furthermore, glioma patients with low CDH2 expression had an improved prognosis and benefited from temozolomide therapy [12]. Similarly, in a thyroid cancer cell line, the downregulation of CDH3 inhibited cell proliferation, migration, and invasion [29].

CDH6 is a transmembrane glycoprotein and a member of the CDH family. Recent studies have shown that CDH6 can be aberrantly overexpressed in cancer. In thyroid cancer, CDH6 expression is strongly associated with EMT, metastatic behavior, and a worse disease outcome [16]. In other malignancies, CDH6 was reportedly associated with tumor growth and a poor prognosis [18, 20]. However, to date, few studies investigating the function of CDH6 in GC exist.

Our research, conducted using multiple public databases as well as GC cell lines and donor-matched GC and healthy gastric tissues, showed that CDH6 was highly expressed in cancerous compared to normal gastric tissues. To analyze the biological functions of CDH6 in GC, GSEA was performed. As shown in Fig. 3a, b, ECM receptor interaction was enriched significantly in CDH6 low-expression groups, which was consistent with several previous bioinformatic studies of patients with GC [30–32]. This result might be related to the decrease in intercellular adhesion and instability of cellular interactions. In addition, *CDH6* was closely associated with genes implicated in energy metabolism such as citrate cycle TCA cycle, glyoxylate and dicarboxylate metabolism, oxidative phosphorylation, and pentose phosphate pathway. In addition, GO analyses suggested that CDH6 might participate in the formation of mitochondrial membrane structures, including the intrinsic component of the mitochondrial inner membrane, mitochondrial matrix, mitochondrial protein complex, and mitochondrial respiratory chain complex assembly.

To determine the diagnostic value of CDH6 in GC, we examined the AUC data. The AUC for TCGA was 0.829, and the AUC for GSE54129 was 0.966, which indicated that the diagnostic efficacy of CDH6 in the context of GC was credible. Conventional biomarkers such as CEA, CA199, and CA72-4, have shown limited diagnostic efficacy for the early detection of GC [33]. However, CDH6 gave a good diagnostic value in the early stages of GC (stage I, AUC = 0.747). Since CDHs have been found not only at the interface between tumor cells but also in bodily fluids (mainly in the blood), CDH6 could be readily detectable in a clinical setting [34]. According to the associations between CDH6 expression and the clinical pathologic features and survival outcome, higher CDH6 levels were found more frequently in GC patients with advanced tumors (at T stage), associated with a poor prognosis. Our univariate and multivariate Cox analyses indicated that the CDH6 expression level was a potential independent marker of poor prognosis in GC. Moreover, survival analyses of the 44 patients with GC recruited from the Tianjin Medical University General Hospital, and Kaplan–Meier plots all supported the same conclusion. In additional, it has been reported that CDH6 could represent a successful therapeutic target for the treatment of ovarian and renal cancers [23]. Our study suggests that CDH6 could be similarly targeted for the treatment of GCs.

In this study, we mainly focused on evaluating the gene expression of CDH6, as well as any associated clinical pathologic features and the survival outcome. Although the Human Protein Atlas (HPA) showed that gene expression of CDH6 was consistent with the protein expression results, further protein and functional experiments need to be performed.

A previous study reported that the expression of CDH6 in oral squamous cell carcinoma was associated with lymph node metastasis and a poor prognosis [20]. In addition, Gugnoni et al*.* reported that CDH6 expression affected the structure and function of mitochondria and promoted EMT and cancer metastasis in the context of papillary thyroid carcinomas [16]. On the contrary, Goeppert and colleagues demonstrated that CDH6 was a putative tumor suppressor and that the downregulation of CDH6 was in fact associated with poor outcome in cholangiocarcinoma patients [35]. These results imply that CDH6 may play divergent roles depending on the tumor type, an intriguing prospect that needs to be further elucidated. According to our analysis, CDH6 expression were associated with improved survival (Additional file 1: Figure S1, Additional file 2: Figure S2). Whether other CDH family members could influence the survival or prognosis of patients with GC needs to be further studied.

## Conclusion

CDH6 was highly expressed in GC, which may represent a potential diagnostic and prognostic GC-specific molecular marker. In addition, high CDH6 expression was significantly associated with a more advanced T stage and poor survival.

## Supplementary Information

**Additional file 1: Figure S1.** The survival analysis of CDH family members in GC.

**Additional file 2: Figure S2.** Cox regression analysis of expression of CDH family members in GC. **a.** The univariate Cox analysis. **b.** The multivariate Cox analysis.

## Data Availability

The datasets generated during the current study are not publicly available since they will contain patient data and the informed consent agreement does not include sharing data publicly. An anonymized form of the data could be made available from the corresponding author upon reasonable request.

## Abbreviations

CDH6: Cadherin 6
GC: Gastric cancer
ROC: Receiver operating characteristic curve
GSEA: Gene set enrichment analysis
TCGA: The Cancer Genome Atlas dataset
AUC: Area under the ROC curve
TCA: Tricarboxylic acid cycle
ECM: Extracellular matrix
GEO: Gene expression omnibus
EMT: Epithelial mesenchymal transition
qRT-PCR: Quantitative real-time-polymerase chain reaction
