# Supplementary material for: High expression of oncogene cadherin-6 correlates with tumor progression and a poor prognosis in gastric cancer
Source: Cancer Cell Int. 2021 Sep 16;21:493. doi: 10.1186/s12935-021-02071-y (PMC8447617; doi:10.1186/s12935-021-02071-y)
Supplement: Supplementary file 1 — Additional file 1: Figure S1. The survival analysis of CDH family members in GC. [file 12935_2021_2071_MOESM1_ESM.pdf]

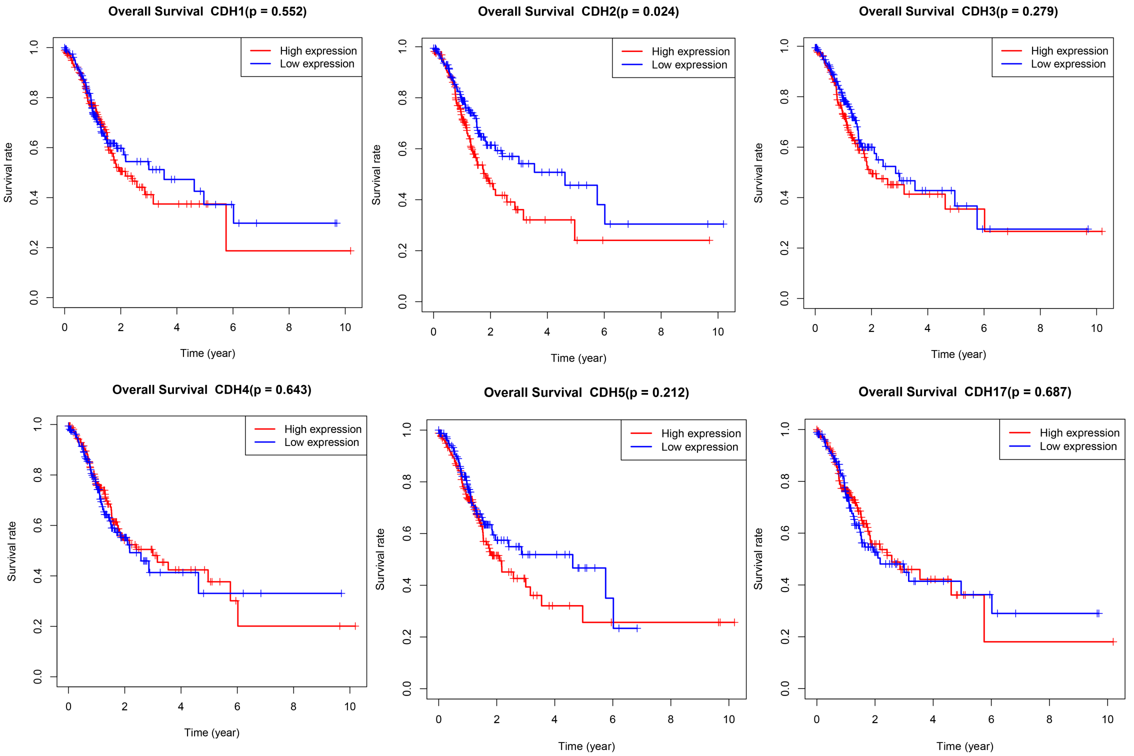
Overall Survival CDH1(p = 0.552)
Overall Survival CDH2(p = 0.024)
Overall Survival CDH3(p = 0.279)
Overall Survival CDH4(p = 0.643)
Overall Survival CDH5(p = 0.212)
Overall Survival CDH17(p = 0.687)
High expression
Low expression
Survival rate
Time (year)
0.0
0.2
0.4
0.6
0.8
1.0
0
2
4
6
8
10
