# Supplementary figures and images for: High expression of oncogene cadherin-6 correlates with tumor progression and a poor prognosis in gastric cancer
Source: Cancer Cell Int. 2021 Sep 16;21:493. doi: 10.1186/s12935-021-02071-y (PMC8447617; doi:10.1186/s12935-021-02071-y)

**a**

## univariate Cox analysis

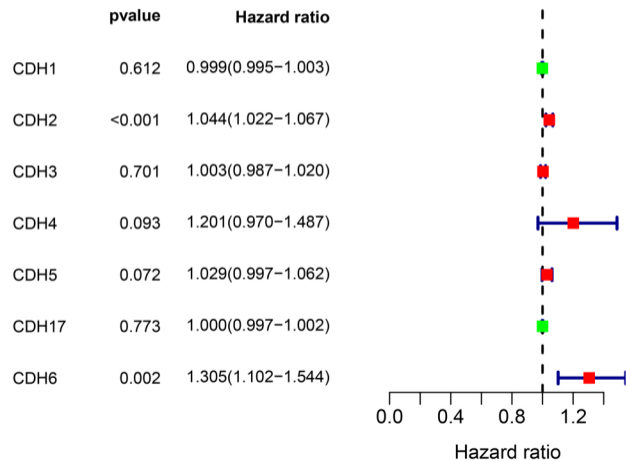**b**

## multivariate Cox analysis

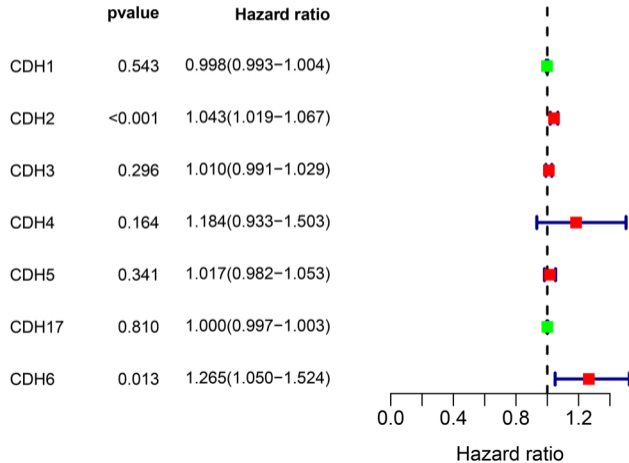

Supplement: Supplementary file 2 — Additional file 2: Figure S2. Cox regression analysis of expression of CDH family members in GC. a. The univariate Cox analysis. b. The multivariate Cox analysis. [file 12935_2021_2071_MOESM2_ESM.pdf]
